# Integrating Prior Authorization Into Clinical Workflows for Care Access and Practitioner Experience

**DOI:** 10.1001/jamanetworkopen.2025.49093

**Published:** 2025-12-22

**Authors:** William C. Chen, Colin Carpenter, Baho Sidiqi, Adam J. Pattison, Jamie Hwang, Damon Pappas, Louis Potters

**Affiliations:** 1 Northwell, New Hyde Park, New York; 2 Department of Radiation Medicine, Northwell Health, Bay Shore, New York; 3 Zucker School of Medicine, Hempstead, New York; 4 Siris Medical, a division of TurningPoint Healthcare Solutions, Lake Mary, Florida; 5 Department of Radiation Medicine, Northwell Health, Lake Success, New York; 6 Department of Radiation Medicine, Northwell Health, New York, New York

## Abstract

**Importance:**

Utilization management strategies such as prior authorization are used to balance appropriate care with cost but can create substantial administrative burdens that can delay timely access to necessary care. A real-time, clinically integrated prior-authorization process may reduce burden and expedite care, especially for complex diseases such as cancer.

**Objective:**

To evaluate the association of clinically integrated prior-authorization software with denial rates, authorization times, and staff satisfaction.

**Design, Setting, and Participants:**

This quality improvement study spanned from January to December 2024, with intervention data collected from August 2023 to December 2024. The study was conducted in a large, multifacility academic radiation practice within a major metropolitan network. Participants included physicians, nurses, and billing staff at the intervention sites. The patient population was covered by 86 health plans, primarily 7 dominant payers (4 national and 3 regional).

**Exposure:**

Three centers implemented the prior-authorization software, while 4 others served as control centers.

**Main Outcomes and Measures:**

The primary outcomes were prior-authorization denial rates and authorization times. Staff satisfaction with the prior-authorization process was assessed through preintervention and postintervention surveys. Denial statistics were compared using the Fisher exact test, and timing statistics were compared using an unpaired *t* test.

**Results:**

Among 6551 (2403 intervention and 4148 historic control) cases from a radiation oncology patient population, implementation of the software was associated with a mean 65.4% reduction in prior authorization denials (from 314 [7.6%] to 63 [2.6%]; *P* < .001) across all payers. Per payer, the denial decrease ranged from 45.7% to 88.6%. Payer and practitioner prescription alignment occurred in 2340 of the 2403 intervention cases (97.4%). The median (IQR) authorization time decreased by 33.9% (from 4.2 [1.7-2.4] to 2.8 [2.4-3.0] business days; *P* < .001), with 18.9% to 52.3% payer-specific reductions; the 90th percentile reported an approximately a 1-week reduction (from 17.7 to 10.5 business days). Surveys of 15 users (7 physicians and 8 billing staff) reported increased satisfaction regarding speed, transparency, ease of use, and overall experience.

**Conclusions and Relevance:**

In this quality improvement study of prior authorization workflows, real-time transparency available at the point of care was associated with reduced rates of denials, reduced delays in care, and improved practitioner satisfaction. These findings suggest that such technology may offer a valuable solution to current challenges in prior authorization by streamlining workflows and facilitating more timely access to care.

## Key Points

**Question:**

Is clinically integrated prior-authorization software associated with improved transparency, reduced administrative burden, and improved patient access to care?

**Findings:**

In this quality improvement study of 6551 cases from a radiation oncology patient population, implementation of a clinically integrated prior-authorization system in a multicenter radiation oncology practice was associated with a statistically significant 65% mean reduction in denial rates, a 34% decrease in median authorization times, and improved staff satisfaction.

**Meaning:**

The findings of this study suggest that real-time transparency at the point of care may enhance patient outcomes with streamlined workflows, reduced costs, and improved timely access to radiation therapy.

## Teaser

This quality improvement study assesses whether clinically integrated prior-authorization software in a multicenter radiation oncology practice is associated with denial rates and authorization times and practitioner experience.

## Introduction

The US health care system grapples with substantial waste,^1^ prompting efforts to implement utilization management strategies such as prior authorization (PA) in an effort to reduce low-value care.^2,3,4,5,6^ While there is evidence that PA can encourage safe, high-value guideline-concordant treatment,^7,8^ traditional processes are often disconnected from clinical workflows, which can impart substantial burdens on health care stakeholders, including clinicians, payers, and patients, often delaying timely access to necessary care, increasing patient anxiety, and potentially leading to poorer treatment outcomes.^9,10,11^

Traditional PA processes are hampered by multiple shortcomings. Two particular issues caused by a disconnected workflow are the lack of transparency regarding medical necessity criteria at the point of care and the lack of automated document retrieval.^12^ The lack of transparency is compounded by considerable variability across payer plans^13^ and criteria that are sometimes divergent from clinical evidence.^14^ Consequently, traditional processes often require multiple iterations of communication with the payer to align on care pathways and transmit the required information.

Specialties such as cardiology, diagnostic radiology, and radiation oncology are disproportionately affected, facing the highest percentage of procedures requiring PA.^11,15^ Radiation oncology faces a particularly heavy burden, despite its high cost-effectiveness; it is used in more than half of cancer cases and accounts for only 3% to 4% of the total yearly cancer care spending.^16,17^

We investigated the outcome of clinically integrating a PA support and automation system into the PA process. We hypothesized that such a system would be associated with an improved authorization process by enhancing transparency of requirements and would be associated with streamlined workflows, reduced denials, shortened authorization times, less administrative burden, and ultimately expedited patient care.

## Methods

### Context and Implementation

This quality improvement study was conducted from January to December 2024, with intervention data collected from August 2023 to December 2024, within a large, multifacility academic practice in a major metropolitan network. Three centers (a suburban community satellite, a primary academic center, and an urban satellite) sequentially implemented the PA software, while 4 other facilities served as control facilities. This study design was chosen due to ease of implementation in a quality improvement setting. Practice characteristics are detailed in Table 1. The Northwell Institutional Review Board waived the requirement for review and approval. Study participants were exempt from informed consent (Common Rule [45 CFR §46]) because the study did not involve the collection of patient outcomes for the purpose of establishing scientific evidence to determine how well an intervention achieves its intended results. This report follows the Standards for Quality Improvement Reporting Excellence (SQUIRE) 2.0 reporting guideline for quality improvement studies.

**Table 1.** Practice Characteristics

Characteristic	Facility, No. (%)		
	All	Main academic site	Satellite centers
Participant			
Locations	7 (100.0)	1 (14.3)	6 (85.7)
Physicians	25 (100.0)	10 (40.0)	15 (60.0)
Disease-site case			
Breast	1616 (24.7)	569 (19.6)	1047 (28.7)
Central nervous system	1001 (15.3)	643 (22.2)	358 (9.8)
Extracranial metastases	802 (12.2)	422 (14.6)	380 (10.4)
Gastrointestinal	397 (6.1)	175 (6.0)	222 (6.1)
Genitourinary	879 (13.4)	290 (10.0)	589 (16.1)
Gynecologic	403 (6.2)	157 (5.4)	246 (6.7)
Head and neck	381 (5.8)	168 (5.8)	213 (5.8)
Hematologic	61 (0.9)	58 (2.0)	3 (0.1)
Lymphoma or myeloma	151 (2.3)	76 (2.6)	75 (2.1)
Musculoskeletal	83 (1.3)	40 (1.4)	43 (1.2)
Sarcoma	76 (1.2)	42 (1.4)	34 (0.9)
Skin	228 (3.5)	81 (2.8)	147 (4.0)
Thoracic	454 (6.9)	169 (5.8)	285 (7.8)
Other and unspecified	19 (0.3)	8 (0.3)	11 (0.3)
Total	6551 (100.0)	2898 (44.2)	3653 (55.8)

The PA process is typically a multistep process, including initiating the authorization request upon treatment-order creation; identifying the relevant payer and gathering necessary documentation; submitting the request to the payer, often involving multiple communication iterations; and receiving and processing the payer’s authorization decision. This process can be complex and time-consuming with each treatment and compounded in specialties with a high volume of PA requests.

### Intervention and Authorization Workflow With PA Support and Automation Software

**Figure 1.**
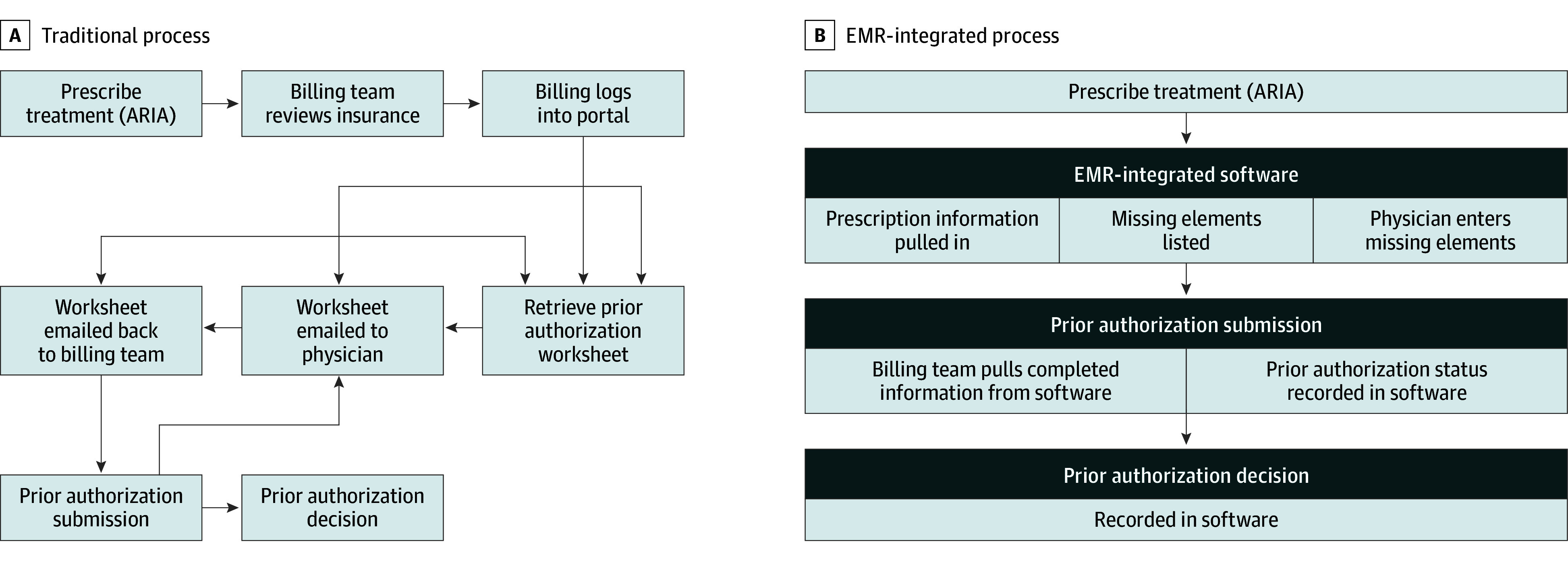
Baseline and Modified Authorization Workflows A, The baseline, traditional authorization process workflow visually conveys with various circular pathways and iterative steps, highlighting its inherent complexity, lack of transparency, and administrative burden associated with its manual, often repetitive, communication loops. B, This is contrasted with the more linear, streamlined modified workflow. ARIA indicates advanced radiation information system; EMR, electronic medical record.

PA support and automation software (InsightRT [Siris Medical, a division of TurningPoint Healthcare Solutions]) was integrated into the clinical workflow. The baseline and modified authorization workflows are depicted in Figure 1. The modified, streamlined workflow leverages the software for the following key steps in the PA process: (1) listening for new treatment orders in the practice management system or electronic medical record to automatically initiate the authorization workflow, (2) identifying the payer and retrieving the necessary documentation for the submission, (3) leveraging the clinical details and providing the clinical team with an assessment against payer policy to predict approval likelihood, and (4) notifying the clinical team of the authorization status. Specifically, the software retrieves specific clinical data elements commonly required by payers for PA submissions, including but not limited to diagnosis codes, staging, additional treatments (eg, prior or future surgery, concurrent systemic therapy), and radiation prescription parameters like technique, fractionation, dose, and image guidance. Missing elements are flagged for manual entry by the clinical staff. The software operates as a rules-based system, not using artificial intelligence, and does not incorporate or predict actual treatment-plan information (eg, dosimetric outcomes, treatment volumes) nor suggest treatment modalities. Its binary approval likelihood analysis is based on aligning collected clinical data with available payer-specific criteria and documentation requirements. Missing data elements or discrepancies alert the clinical team to needs for additional information, which may result in delays.

Physician practice staff (billers, nurses, and physicians) at intervention sites received brief training on the use of the software. All treatments requiring PA at intervention sites, encompassing commercial, Medicare Advantage, Medicaid, and government payers, were processed through the software. Traditional Medicare cases not requiring PA were automatically flagged within the software as *prior authorization not required*. The study patient population was covered by 86 health plans, with the majority covered by 7 dominant plans (4 national and 3 regional), denoted in this study as payers A to G.

### Data Collection Methods

All denial and timing data were recorded in the electronic medical record per standard practice. Denials were identified in the medical record by a task indicating that communication was needed with the payer. The time to authorization was measured in business days, calculated as the number of business days between the initial prescription creation and the recorded payer authorization. Baseline denial data were collected from January 2021 to December 2024; intervention denial data covered August 2023 (the first practice to go live) to December 2024. Timing data were collected from December 2022 to December 2024 (timing data were unavailable before December 2022). Data were binned by payer for analysis to produce resulting statistics for the 7 major payers in the market, as well as for all payers.

Staff satisfaction was assessed using an online Google Forms survey distributed in March 2024, several months after implementation to ensure adequate user experience. The survey targeted all eligible staff directly engaged in the intervention PA process (10 clinical and 8 billing staff using the software). Participants rated (1-5 scale, 5 = best) the following questions: (1) “How would you rate the speed of the prior authorization process before/after the [automation] software was introduced into the clinic?” (2) “How would you rate the transparency for knowing whether a treatment is in [payer] policy before/after the [automation] was introduced into the clinic?” (3) “How would you rate the ease of prior authorization before/after the [automation] software was introduced into the clinic?” (4) “How would you rate the prior authorization experience before/after the [automation] software was introduced into the clinic?” Users also rated (1-10 scale, 10 = best) the question: “How likely is it that you would recommend the software to a colleague?”

### Statistical Analysis

Data analyses were performed using the SciPy toolkit, version 1.15.3 and the statsmodels toolkit, version 0.14.5 (Python Software Foundation). Denial rates were computed based on the total cases experiencing a denial vs the total cases submitted for PA for each major payer and for all payers. Timing data were compared, analyzing the median, the 75th percentile, and the 90th percentile ordered on the length of time to authorization; data were computed for each major payer and for all payers. To investigate whether the software was associated with physician-prescribing patterns, use of the prescribed treatment modalities was compared presoftware and postsoftware implementation for all practices that adopted the software. Denial statistics were compared using the Fisher exact test; timing statistics were compared using an unpaired *t* test; and prescribing patterns were aggregated for all practices before implementation and compared with aggregated patterns after implementation using a χ^2^ test. Statistical significance was defined as 2-sided *P* < .05.

## Results

Among 6551 total cases from a radiation oncology patient population, 2403 were submitted using the automated platform and compared with 4148 historic control cases across all payers. Use of the clinically integrated authorization tool significantly decreased initial PA denials across all payers. Overall denials were reduced by a mean of 65.4% (from 314 [7.6%] to 63 [2.6%]; *P* < .001) (Table 2). More specifically, across payers A to G, baseline denials ranged from 3.9% to 21.2%. With the software, these denials ranged from 1.5% to 6.3%, a reduction ranging from 45.7% to 88.6%. PA denials were appealed at the physician’s discretion. Ultimately, final treatment prescription was aligned in 2340 of 2403 cases (97.4%). Comparing aggregated software users’ prescribing patterns for major treatment modalities before and after implementation showed no statistically significant difference in utilization (eTable 1 in Supplement 1).

**Table 2.** Summary of Prior Authorization Outcomes

Outcome	Baseline			After software implementation			% Reduction	*P* value
	No.		Denial rate, %^a^	No.		Denial rate, %^a^		
	Total	Denials		Total	Denials			
Overall	4148	314	7.6	2403	63	2.6	65.4	<.001
By payer								
A	571	56	9.8	178	8	4.5	54.2	.03
B	238	43	18.1	95	6	6.3	65.0	.01
C	386	33	8.5	194	9	4.6	45.7	.09
D	209	43	20.6	112	4	3.6	82.6	<.001
E	90	12	13.3	66	1	1.5	88.6	.01
F	132	28	21.2	27	1	3.7	82.5	.03
G	441	17	3.9	377	6	1.6	58.7	.06
By location								
Academic	1454	121	8.3	1444	20	1.4	83.4	<.001
Satellite	2694	193	7.2	959	43	4.5	37.4	.01
By disease site								
Breast	1062	73	6.9	554	12	2.2	68.5	<.001
Central nervous system	405	21	5.2	596	20	3.4	35.3	.19
Extracranial metastases	566	41	7.2	236	8	3.4	53.2	.04
Gastrointestinal	324	44	13.6	73	1	1.4	89.9	.10
Genitourinary	594	47	7.9	285	6	2.1	73.4	<.001
Gynecologic	248	27	10.9	155	8	5.2	52.6	.07
Head and neck	232	10	4.3	149	4	2.7	37.7	.58
Hematologic	20	2	10.0	41	0	0	100.0	.10
Lymphoma or myeloma	92	8	8.7	59	2	3.4	61.0	.32
Musculoskeletal	53	3	5.7	30	0	0	100.0	.55
Sarcoma	62	6	9.7	14	1	7.1	26.2	1.00
Skin	149	7	4.7	79	0	0	100.0	.10
Thoracic	329	24	7.3	125	1	0.8	89.0	.01
Other and unspecified	12	1	8.3	7	0	0	100.0	1.00

^a^ Denial rates compare baseline workflow with InsightRT software implementation.

**Figure 2.**
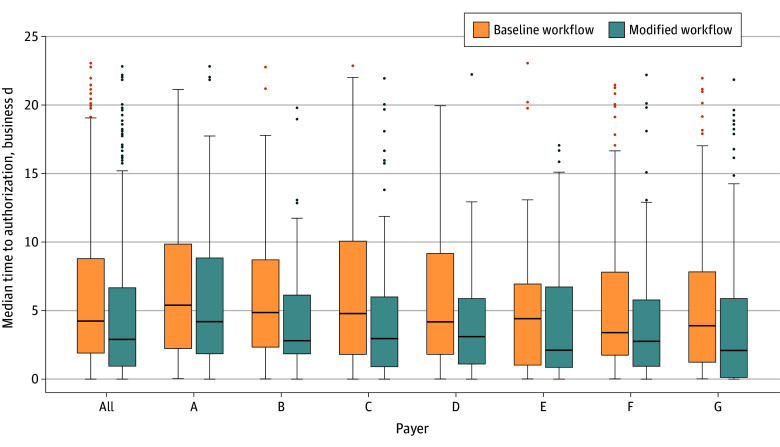
Timing Statistics of Days to Authorization for Baseline and Modified Workflows Horizontal lines inside boxes indicate medians; outer horizontal box lines, IQRs; whiskers, ranges; circles, outlier data.

Significant reductions were also observed in the median time to authorization across all payers. The overall median (IQR) time to authorization decreased by 33.9% (from 4.2 [1.7-2.4] to 2.8 [2.4-3.0] business days; *P* < .001) (Table 2). Payers A to G had reductions in the median time to authorize of 18.9% to 52.3%, from a baseline median (IQR) time of 3.4 [1.8-7.8] to 5.4 [2.2-9.8] business days to a reduced time to authorize of 2.1 [1.0-6.9] to 4.2 [2.2-9.8] business days. Similar reductions were found in the 75th percentile by 24.2% across all payers (2.1 business days); payers A to G had a reduced 75th percentile time to authorize from 3.1% to 40.2%, realizing a reduction in time to authorize from 6.9 to 10.0 business days to 5.8 to 8.8 business days. Also significant was the reduction in the cases that had the longest delays to authorize—the 90th percentile. This population had a reduction of 24.4% (3.9 business days); across payers A to G, the 90th percentile reduced from 11.7 to 17.7 business days to 10.5 to 15.2 business days, with the largest difference being 6.0 business days (payer C). Full details of the timing statistics are shown in Figure 2 and in eTable 2 in Supplement 1.

**Figure 3.**
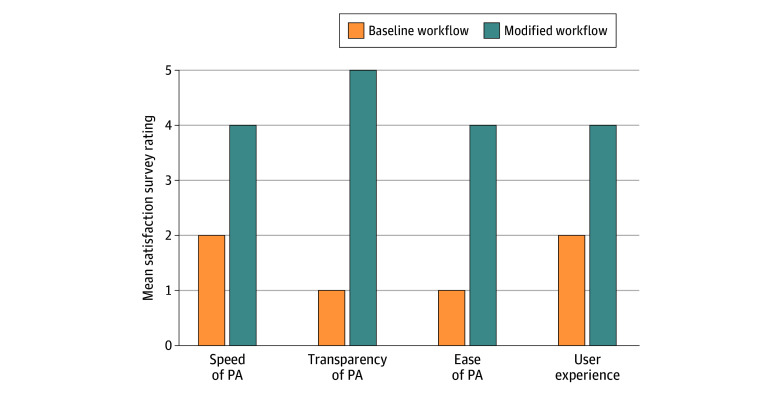
User Satisfaction Survey Results Mean ratings were on a 1-to-5 scale, with 5 being the best. PA indicates prior authorization.

A total of 15 users completed the survey, comprising 7 physicians and 8 billing staff, representing an overall response rate of 83.3% (70% clinical, 0% nurses, and 100% billing staff). Physician ratings significantly improved across all survey domains. Mean (SD) satisfaction scores increased for speed (from a rating of 2 [1] to 4 [1]), transparency (from 1 [1] to 5 [1]), ease of use (from 1 [1] to 4 [1]), and user experience (from 2 [1] to 4 [1]) on a 5-point scale. The results are detailed in Figure 3. Mean (SD) scores for likelihood of recommendation to a colleague increased from 3 (2) for the standard PA workflow to 8 (2) for the modified software-based workflow on a 10-point scale. Surveys across billing staff showed more modest improvements, although satisfaction scores did improve for speed (from 3.0 to 4.0), transparency (from 2.5 to 4.0), ease of use (from 3.0 to 4.0), user experience (from 3.5 to 4.0), and likelihood of recommendation to a colleague (from 4.5 to 8.0).

## Discussion

The burden of traditional PA may substantially impact practitioners, particularly in high-cost care settings. While utilization management strategies such as PA aim to curb health care costs,^18^ they may impose substantial time demands on physicians and staff. A 2024 survey by the American Medical Association found that physicians and their staff spend an average of 12 hours per week managing PA requests, with each physician completing approximately 43 authorizations.^19^ Further emphasizing a lost opportunity, a physician-directed survey of 300 oncology practitioners indicated that time and resources devoted to PA could otherwise be used to increase the number of patients treated, advance research, and improve supportive services.^20^ A national survey of both medical oncology and radiation oncology trainees found that 40% spent over 30 minutes on each PA appeal, with more than 75% of appeals overturned by the payer, highlighting process inefficiency.^21^

This administrative burden can contribute to burnout; a recent survey from the American Society for Radiation Oncology noted that more than 90% of radiation oncologists reported worsened staff burnout in their cancer clinics because of PA.^22^ Burnout may not only negatively impact staff well-being but also may carry financial implications through increased workforce attrition, reduced productivity, and costs associated with recruiting and training replacements.

The financial costs of administering PA can be substantial. Estimates suggest that the time spent by physicians, nursing, and clerical staff on PA-related administrative tasks costs more than a $68 000 time equivalent per physician per year.^23^ Within radiation oncology, Bingham et al^24^ reported that PA costs range from $27 to $100 per patient, depending on the need for peer-to-peer review.

Traditional PA processes can negatively impact patients, primarily through treatment delays. In radiation oncology, 68% of physicians report average delays to patient treatment of 5 days or longer, often necessitating initiation of less optimal treatment approaches (82% of respondents).^22^ The data in our study showed that even with software implementation, authorization times at the 90th percentile still reached up to 15.2 business days, equating to approximately 3 calendar weeks. Such delays substantially challenge best practices in oncology, as tumor growth or anatomic changes during this waiting period undermine the accuracy and effectiveness of precise modern radiation treatments. These delays are perceptible to the patient, with 69% noting PA-related delays that may lead to increased anxiety and reduced trust of their insurance company and the health care system.^25^

Similar delays are noted in other specialties. Shah et al^26^ reported PA delays of 7 days for patients with inflammatory bowel disease who were prescribed biologics, extending to 29 days when an appeal was required, which correlated with poorer clinical outcomes. Lizcano et al^27^ similarly reported significant mean (SD) delays averaging nearly 1 month for knee (total knee arthroplasty, 26.3 [34.6] days) and hip (total hip arthroplasty, 33.7 [41.5] days) orthopedic procedures. A similar burden has been reported in other specialties, including adult cardiology^28^ and pediatric cardiology.^29^

These findings underscore the need for streamlined PA processes that minimize burdens on both practitioners and patients. The Centers for Medicare & Medicaid Services (CMS) Interoperability and Prior Authorization Final Rule (Final Rule),^30^ published in February 2024, mandates improved standards for process efficiency, timing, and transparency. For the present study, our implemented technologic platform specifically supported the practitioner-to-payer provisions of improved transparency and data exchange, demonstrating meaningful opportunity from this initiative to reduce PA burden.

Other strategies to address the PA process are ongoing, with different methods being attempted. One high-profile strategy involves gold-carding practitioners, which would provide exemptions to those who historically have very high approval rates. While no threshold is publicly shared for gold-carding status, use of the clinically integrated authorization tool in our study was able to decrease PA denial rates to less than 3%.

Part of this improvement may be associated with improved transparency of the PA process. The opacity of payer medical necessity criteria often leads to inadvertent omissions in practitioner submissions, particularly for high-cost, complex cases.^29^ Complex care necessitates extensive documentation to demonstrate medical necessity. In oncology, for instance, PA submissions may require detailed patient information beyond an oncologic diagnosis, including cancer type and stage, clinical documentation of the patient’s condition, and the specific characteristics of a proposed treatment plan. Having the missing data elements flagged for the clinical team allows upfront retrieval before submission, hopefully satisfying the benefit managers’ query.

Notably, we observed disparate denial rates across payers, even for treatments widely accepted as standard of care. This variability highlights the often subjective or inconsistent nature of PA practices and underscores the critical need for greater transparency and standardization in payer policies, reinforcing the value proposition of technologic interventions designed to help meet the objectives outlined in the CMS Final Rule. Transparency of requested data elements was also associated with improved perceived speed of PA data collection and submission, among both clinicians and billing staff, with appreciable reductions in median time to authorize treatments.

### Limitations

This study has limitations. It was conducted within a single, large academic medical center network, and results may not be generalizable to smaller practices or different health care settings. The variability in payer policies and the adoption of electronic PA systems may influence the effectiveness of automated solutions. Further research across diverse payer models and practice settings is warranted to confirm the generalizability of these findings.

Given the quality improvement nature of this study and small number of intervention centers, we incorporated historical controls and conducted this study over an aggregate intervention period rather than in a formal, randomized stepped-wedge design. A stepped-wedge framework may have strengthened causal inference. Future work should address generalizability through a more formal analysis. Although the study did not randomize by site of care, a series of statistical robustness checks validated significant reductions in authorization time and denial frequency associated with the software intervention that remained when accounting for site.

We acknowledge the ethical concern that automated PA systems, particularly when payer policies may not fully align with evolving evidence-based guidelines, could inadvertently influence physician prescribing or discourage medically indicated treatments. However, our system was designed to display real-time payer-specific policy requirements for the physician’s chosen treatment plan, not to dictate clinical decisions. Importantly, analysis of the study’s intervention arm showed no statistically significant change in the proportion of treatment modalities prescribed, suggesting that the tool did not overtly steer clinical decisions. Ultimately, such systems aim to reduce administrative friction and facilitate timely access to medically appropriate care, without compromising clinical judgment.

Our approach to automating the process is not singular in its effort; automation is also the focus of a standards-based approach in Health Level 7 International’s Da Vinci Fast Healthcare Interoperability Resource accelerator program.^12,31^ Advances in artificial intelligence technologies may offer future integration and opportunities for increased automation.^32^ And further integration into payer workflows would likely offer additional speed improvements. However, broader adoption of automation faces potential barriers, including the initial costs of software and implementation, staff training requirements, and ensuring payer compliance with automated systems.

## Conclusions

In this quality improvement study of PA workflows, clinically integrating PA support software was significantly associated with reduced denials and authorization times, improved staff satisfaction, and expedited patient access to necessary care, which may have ultimately enhanced the value of care delivery. This streamlined workflow was associated with greater efficiency between clinical staff and revenue-cycle operations, working more seamlessly toward initiating patient care. In an era of increasing digitization and administrative automation, this method may be far more scalable. Broader implementation of such connected systems has the potential to alleviate the pervasive challenges of PA in health care, particularly in areas of complex care, but will require further integration with national payer policies and standardized data exchange to maximize its benefits. This approach not only may provide immediate improvements but also sets the stage for future research assessing long-term impacts on health care costs and patient outcomes, ensuring access to timely, high-quality care.

## Notes

Supplement 1. **eTable 1.** Summary of Prescribing Patterns Preimplementation and Postimplementation of the Software for Practices That Used the Software **eTable 2.** Time to Authorization, Measured in Business Days

Supplement 2.
Data Sharing Statement
