# Supplementary material for: Integrating Prior Authorization Into Clinical Workflows for Care Access and Practitioner Experience
Source: JAMA Netw Open. 2025 Dec 22;8(12):e2549093. doi: 10.1001/jamanetworkopen.2025.49093 (PMC12723545; doi:10.1001/jamanetworkopen.2025.49093)
Supplement: Supplement 1. — eTable 1. Summary of Prescribing Patterns Preimplementation and Postimplementation of the Software for Practices That Used the Software eTable 2. Time to Authorization, Measured in Business Days [file jamanetwopen-e2549093-s001.pdf]

Supplemental Online Content

Chen WC, Carpenter C, Sidiqi B, et al. Integrating prior authorization into clinical workflows for care access and practitioner experience. *JAMA Netw Open*. 2025;8(12):e2549093. doi:10.1001/jamanetworkopen.2025.49093

eTable 1. Summary of Prescribing Patterns Preimplementation and Postimplementation of the Software for Practices That Used the Software
eTable 2. Time to Authorization, Measured in Business Days

This supplemental material has been provided by the authors to give readers additional information about their work.

© 2025 Chen WC et al. *JAMA Network Open*.

eTable 1. Summary of Prescribing Patterns Preimplementation and Postimplementation of the Software for Practices That Used the Software

	3D% pre	3D% post	IMRT% pre	IMRT% post	SBRT% pre	SBRT% post	Other pre	Other post
Practice 1	20%	33%	50%	40%	16%	6%	14%	21%
Practice 2	63%	27%	13%	39%	0%	24%	24%	10%
Practice 3	25%	28%	75%	30%	0%	19%	0%	23%
Practice 4	3%	3%	38%	37%	51%	52%	8%	8%
Practice 5	52%	50%	34%	32%	7%	6%	7%	12%
Practice 6	64%	61%	16%	20%	8%	9%	12%	10%
Practice 7	4%	2%	69%	62%	27%	33%	0%	3%
Practice 8	41%	56%	34%	27%	11%	6%	14%	11%
Practice 9	36%	22%	27%	22%	29%	48%	8%	8%

© 2025 Chen WC et al. *JAMA Network Open*.

eTable 2. Time to Authorization, Measured in Business Days

	All	Payer A	Payer B	Payer C	Payer D	Payer E	Payer F	Payer G
**Baseline cases**	1583	314	163	149	226	51	283	266
**InsightRT cases**	2403	178	95	194	112	66	27	377
**Baseline time to authorization, median (days)**	4.2	5.4	4.9	4.8	4.2	4.4	3.4	3.9
**InsightRT time to authorization, median (days)**	2.8	4.2	2.8	3.0	3.1	2.1	2.8	2.1
**Percent reduction, median (%)**	33.86%	22.26%	42.32%	38.21%	25.75%	52.30%	18.90%	46.28%
**Baseline time to authorization, 75th percentile (days)**	8.8	9.8	8.7	10.0	9.1	6.9	7.8	7.8
**InsightRT time to authorization, 75th percentile (days)**	6.7	8.8	6.1	6.0	5.9	6.7	5.8	5.8
**Percent reduction, 75th percentile (%)**	24.21%	10.27%	29.76%	40.20%	35.88%	3.14%	25.96%	25.23%
**Baseline time to authorization, 90th percentile (days)**	15.9	17.7	15.1	16.9	16.3	11.7	12.2	14.2
**InsightRT time to authorization, 90th percentile (days)**	12.0	15.2	12.9	10.9	10.9	12.8	10.5	12.0
**Percent reduction, 90th percentile (%)**	24.45%	14.19%	14.83%	35.68%	32.85%	-8.92%	14.20%	15.54%
**p-value**	0.00	0.78	0.19	0.01	0.05	0.95	0.42	0.03

© 2025 Chen WC et al. *JAMA Network Open*.
