# Supplementary material for: Integrating Prior Authorization Into Clinical Workflows for Care Access and Practitioner Experience
Source: JAMA Netw Open. 2025 Dec 22;8(12):e2549093. doi: 10.1001/jamanetworkopen.2025.49093 (PMC12723545; doi:10.1001/jamanetworkopen.2025.49093)
Supplement: Supplement 2. — Data Sharing Statement [file jamanetwopen-e2549093-s002.pdf]

Data Sharing Statement

Chen,. Integrating Prior Authorization Into Clinical Workflows for Care Access and Practitioner Experience. *JAMA Netw Open*. Published December 22, 2025.
doi:10.1001/jamanetworkopen.2025.49093

Data
Data available: No
